# Exercise in Sickle Cell Disease: The Impact on Pathophysiology and Clinical Symptoms

**DOI:** 10.3390/children13070849

**Published:** 2026-06-24

**Authors:** Charlie Gill, Anne Greenough, James Cook

**Affiliations:** 1 Neonatal Intensive Care Unit, King’s College Hospital, 4th Floor Golden Jubilee Wing, Denmark Hill, London SE5 9RS, UK; charlie.gill1@nhs.net; 2 Department of Women and Children’s Health, School of Life Course Sciences, Faculty of Life Sciences and Medicine, King’s College London, London SE5 9RS, UK; 3 Paediatric Respiratory Department, King’s College Hospital, Denmark Hill, London SE5 9RS, UK; jwa.cook@nhs.net

**Keywords:** sickle cell disease, exercise, physical activity, vaso-occlusive crisis, pain, inflammation, vascular dysfunction, blood rheology, oxidative stress, guidelines

## Abstract

**Highlights:**

**What are the main findings?**

- Numerous studies have reported encouraging safety data for brief periods of high-intensity exercise, extended periods of moderate-intensity exercise, and regular exercise training programmes of moderate intensity conducted over eight to twelve weeks for patients with sickle cell disease.
- Regular physical activity can potentially have a positive impact on numerous underlying pathophysiological processes in sickle cell disease.

**What are the implications of the main findings?**

- The impact of regular exercise training in sickle cell disease represents an exciting area for future research, with a particular need to investigate how regular physical activity impacts patients’ clinical conditions and outcomes.
- Focus should be on identifying an ideal level of exercise that provides maximum benefit for minimum risk and in developing a set of evidence-based generalised exercise guidelines and recommendations for the sickle cell disease population.

**Abstract:**

Sickle cell disease (SCD) is one of the most common inherited blood disorders worldwide. Clinical manifestations are variable, but include hyposplenism, renal impairment, cardiovascular disease, respiratory complications, and cerebrovascular disease. Frequent painful vaso-occlusive crises, hospitalisations, and other physical and psychological ramifications can have profound effects, including children missing school time resulting in impaired academic performance and adults missing work leading to employment loss. This narrative review examines the possible risks and benefits of exercise in the SCD population. Regular exercise plays an important role in improving physical and mental health, but fears around the potential consequences of exercise for the SCD population are present in children, their families, schools, and other organisations. This can result in children not taking part in as much regular exercise as their peers and being excluded from group activities. Studies have suggested that healthcare professionals often do not discuss the possible benefits of physical exercise with patients, likely because there are no guidelines regarding a safe level of activity. An acute increase in inflammation secondary to exercise could increase the risk of vaso-occlusive crises, but regular physical activity is known to play an important role in disrupting chronic inflammation across a wide range of pro-inflammatory diseases. Indeed, studies have demonstrated positive responses to exercise in the SCD population, from improvements in skeletal muscle microvasculature to performance in cardiovascular tests. It is important that recommendations are developed regarding types of exercise and the ideal amount of exercise for maximum benefit with minimum risk in SCD individuals.

## 1. Introduction

Sickle cell disease (SCD) is one of the most common inherited blood disorders worldwide. Based on an estimated birth rate of more than 300,000 each year, it is thought that at least three million people are living with the condition [1]. About 75% of these are in sub-Saharan Africa [2]. Sickle cell disease is an autosomal recessive genetic disorder where a single nucleotide mutation (adenine to thymine) in the β-globin gene results in the substitution of valine for glutamic acid and the production of HbS—sickle haemoglobin [3]. Under deoxygenated conditions, haemoglobin polymerisation occurs due to the hydrophobic residue of the valine binding to other hydrophobic residues, causing HbS molecules to aggregate [4]. This polymerisation results in the formation of long branching chains of HbS that can disrupt the architecture and flexibility of the erythrocyte, leading to the formation of characteristic “sickle-shaped” red blood cells, which become rigid, fragile, and can obstruct vessels. Cellular dehydration also results, further contributing to the stress on the cell. The polymerisation of haemoglobin, resulting in dehydrated sickled erythrocytes, leads to two major pathophysiological processes—vaso-occlusion with associated ischaemia–reperfusion injury and intravascular haemolysis and haemolytic anaemia [3]. Over time, this leads to progressive organ damage.

The formation of dehydrated, deformed, and rigid sickled erythrocytes predisposes to the occlusion of post-capillary venules as a result of their interaction with leucocytes and the vascular endothelium. Vaso-occlusion in turn leads to infarction, haemolysis, and inflammation. An increase in inflammation and the levels of pro-inflammatory cytokines, including IL-1β, IL-6, IL-8, and TNF-α, results in endothelial activation and drives increased expression of VCAM-1 (vascular cell adhesion molecule-1), ICAM-1 (intracellular adhesion molecule-1), and other adhesion molecules. This further increases the tendency of sickled erythrocytes to adhere to the vascular endothelium and precipitate vaso-occlusive events in a vicious cycle [5,6,7,8]. Restoration of blood flow to the post-capillary venules then results in reperfusion-mediated injury that generates free radicals and causes oxidative damage, resulting in further inflammation, vasculopathy and endothelial dysfunction. The haemolytic anaemia seen in SCD is also driven by HbS polymerisation, which results in the formation of sickled erythrocytes that are rigid and fragile. The intravascular haemolysis seen also contributes to endothelial dysfunction as a result of an increase in free haemoglobin in the plasma. Free plasma haemoglobin, as well as free heme, generates free reactive oxygen species (ROS). ROS damage endothelial cells directly through peroxidation of the lipid membrane and DNA fragmentation, and also strongly bind to nitric oxide (NO) [9,10,11]. The functional NO deficiency that results further contributes to the vasculopathy, with increased expression of adhesion molecules that NO usually inhibits, such as VCAM-1 and ICAM-1 [12]. This further plays into the vicious cycle by contributing to an increased risk of adhesion between sickled erythrocytes and the endothelium and ultimately an increased risk of vaso-occlusive events. Release of arginase-1 into the plasma as erythrocytes haemolyse also plays a role. Arginase metabolises arginine in the plasma into ornithine and, in doing so, decreases the required substrate for endothelial NO synthesis. This further contributes to the decreased NO bioavailability seen in SCD [13,14]. Markers of oxidative stress have been found to have a significant association with the loss of microvascular function in children with SCD [15], and an association has been found between plasma free heme concentration and the incidence of vaso-occlusive crises and ACS in children with SCD [16]. There is also evidence that haemolytic products, including free plasma haemoglobin and heme, act as erythrocytic DAMPs (danger-associated pattern molecules) and can further contribute to the chronic inflammation and vasculopathy seen in SCD through directly activating innate immune pathways via toll-like receptor 4 (TLR4) and NOD-like receptor pyrin domain containing 3 (NLRP3) inflammasome signalling [11,17]. Oxidative stress likely also has direct effects on red blood cell (RBC) rheology, which is known to be abnormal in SCD patients [4,18]. Studies on the response of RBCs to oxidative stress have shown a decrease in RBC deformability, a slight decrease in RBC aggregation and a large increase in the strength of RBC aggregates (the sheer stress required to disperse RBC aggregates that have formed) [19,20]. Decreased RBC deformability has itself been shown to result in a decrease in tissue oxygenation and blood flow in the microcirculation [21,22], and an association between increased RBC aggregate strength and acute chest syndrome (ACS) in children with SCD has been demonstrated [23].

SCD confers a significant burden on patients, with reduced average life expectancy [24] and a significant negative impact on quality of life [25,26]. There is a large variation in the clinical manifestations and outcomes of SCD, which vary from death in childhood to a relatively symptom-free life into the eighth decade [1]. A large study in the US of over 3500 SCD patients showed that while there was an average of 0.8 significant episodes of pain a year, nearly 40% of patients had no episodes, and just over 5% of patients had between three and ten episodes a year [27]. A study of cerebrovascular disease in over 200 children with SCD showed that 50% of patients have some form of cerebrovascular disease by the age of 14, with the other half showing no evidence of this problem [28]. The extent of this variability remains unexplained. Clinical manifestations are widespread and variable, but include hyposplenism, renal impairment, cardiovascular disease, respiratory complications, and cerebrovascular disease [29]. Sickle cell disease has a huge impact on the individual’s quality of life. Frequent painful vaso-occlusive crises, hospitalisations, and other physical and psychological ramifications can have profound effects, including children missing school time, resulting in impaired academic performance [30], and adults missing work, leading to employment loss and financial difficulties [31].

Regular aerobic exercise plays a role in sustaining and improving physical and mental health and wellbeing [32,33]. On the contrary, a lack of physical activity increases all-cause mortality and overall poor health [32,33,34,35,36] and is associated with the development of a wide range of chronic health conditions [37]. The role of regular exercise in providing protection against, and potentially treatment for, a wide variety of chronic diseases is increasingly clear. Regular exercise has been demonstrated to have protective effects against cardiovascular disease, type 2 diabetes mellitus, colon cancer, and breast cancer, for example [38]. Despite this, patients with SCD have historically often felt discouraged from participating in physical activity, whether secondary to their own fears of experiencing a vaso-occlusive crisis [39,40], the fears of their parents or their schools, or the fears of healthcare professionals. This may be due to the lack of guidelines for exercise in this population [41]. Patients with SCD are more likely to be sedentary [42] and have reduced physical fitness with decreased exercise capacity and tolerance compared to healthy controls [43,44]. It has been feared that changes induced by acute exercise, such as acidosis, oxidative stress, and dehydration, may increase the risk of sickling and acute clinical complications. There is, however, a growing body of evidence that moderate intensity exercise training is safe and well tolerated by SCD patients, and may confer a number of health benefits, including improved cardiorespiratory function and muscular function [17]. This narrative review examines the possible risks and benefits of exercise in the SCD population.

## 2. Methods

A literature search was conducted in PubMed for studies and reviews related to sickle cell disease and exercise. The search strategy involved searching separately for reviews on the pathophysiology of sickle cell disease, for studies and reviews of both sickle cell disease and exercise together (using the search terms of “sickle cell” and “exercise”), and then a number of more specific searches on the impacts of exercise on inflammation, vascular dysfunction, blood rheology, NO metabolism and oxidative stress, cardiovascular and respiratory function, and on muscle structure and function in healthy subjects or other disease populations. All studies found during this literature search that involved patients with SCD undertaking either acute bouts of exercise or regular exercise training programmes were included.

## 3. Exercise and Sickle Cell Disease

### 3.1. Reduced Exercise Tolerance

A major contributor to the reduced exercise capacity seen in SCD individuals is chronic anaemia. A small study of ten patients with SCD has shown that a single partial exchange transfusion can improve the exercise capacity of SCD patients [45]. This may be in part due to the increase in haemoglobin, but other factors must be contributing, as large increases in exercise capacity were seen for relatively modest improvements in haemoglobin. Furthermore, two of the patients had a reduction in haemoglobin levels following the transfusion but were still found to have their exercise capacity improve. In addition to anaemia, abnormal blood rheology is likely to play a role in reduced exercise tolerance in SCD patients. RBC deformability has been demonstrated to be an independent predictor of six-minute walking test performance in children with SCD, alongside haemoglobin levels [46].

Cardiac dysfunction is also likely to play a role in the reduced exercise capacity seen in the SCD population. Chronic anaemia leads to an increase in cardiac output secondary to increases in left ventricular stroke volume, with only minimal increases in heart rate, and this results in significant dilation of the left ventricle [47]. Over time, this progressive dilation results in increased wall stress, increased left ventricular mass, and diastolic dysfunction. Diastolic dysfunction has been shown to be common in children with SCD [48,49], and to correlate with reduced exercise tolerance [50,51]. The degree of diastolic dysfunction is independently associated with early increases in blood lactate concentrations during exercise [52]. Earlier increases in blood lactate for a given work rate and oxygen consumption (VO2) suggest lower physical fitness.

SCD is also associated with pulmonary dysfunction. Lung function abnormalities are frequently noted in children and adolescents with SCD and lung function declines with increasing age [53,54]. Pulmonary hypertension is a serious and common complication of SCD, seen more in the adult than the paediatric population [55,56]. A history of recurrent ACS and restrictive lung disease has been associated with reduced exercise capacity and impaired performance in a six-minute walking test in children and young adults with SCD [57]. Gas-exchange data obtained from cardiopulmonary exercise testing (CPET) in children and adults with SCD shows impaired O_2_ uptake, reduced ventilatory efficiency, and a lower O_2_ pulse (the amount of oxygen consumed per heartbeat) during exercise when compared to healthy controls [44,58,59,60].

Muscular dysfunction also plays a role in contributing to limited aerobic capacity in SCD, as well as decreased anaerobic capacity and muscle strength [43,61,62]. Patients with SCD show skeletal muscle hypotrophy [63,64,65], and histological analysis reveals reduced capillary density and decreased capillary tortuosity [64,65]. Skeletal muscle in SCD patients has also been found to display impaired oxidative capacities, with lower oxidative enzyme activity than healthy controls [63,64]. This lower oxidative capacity leads to an earlier favouring of non-oxidative glycolysis and an earlier accumulation of lactate during exercise in SCD patients [59,66,67]. It is unclear to what degree these changes are direct pathophysiological consequences of SCD versus deconditioning secondary to the decreased level of physical activity seen in individuals with SCD when compared to healthy controls [42,68].

### 3.2. Concerns Regarding Acute Exercise

The concerns around acute exercise in SCD have largely centred around fears that it will increase the risk of sickling and hence the risk of vaso-occlusive crises, particularly at high intensities. Exertion has frequently been noted as a potential precipitant of vaso-occlusive crises in patient-reported questionnaires [69]. For example, one study found that 30% of patients presenting to the emergency department with a severe vaso-occlusive crisis requiring hospitalisation reported exertion as a precipitating factor [70].

Acutely, exercise results in an inflammatory response characterised by the mobilisation of leukocytes and increased levels of circulating inflammatory mediators [71,72]. The exact form of inflammation varies according to the type, intensity, and duration of any exercise, as well as the relative fitness of the participant. SCD causes chronic inflammation, with raised pro-inflammatory mediators (including IL-1, IL-6, IL-8, and TNFα) found at rest in SCD patients when compared to healthy controls [8,73,74,75]. Given the role inflammation plays in the pathophysiology of SCD and the already higher baseline inflammatory state in these patients, further increases in the level of inflammation during and immediately following an acute bout of exercise could further drive the expression of vascular endothelial adhesion molecules and further increase the risk of sickled RBCs adhering to the endothelium and precipitating vaso-occlusive events. Indeed, intense exercise in healthy individuals has been shown to increase levels of endothelial adhesion molecules, including ICAM-1, VCAM-1, and some selectins [76].

During moderate to intense exercise, there is a reduction in pH due to the production of lactic acid by anaerobic respiration. This decreases the affinity of haemoglobin to oxygen through the Bohr effect, which is usually beneficial to facilitate the delivery of oxygen to the tissues. In the context of SCD, however, the increased deoxygenation could precipitate HbS polymerisation and a vaso-occlusive event [17]. Acidosis has also been shown to be an independent risk factor for HbS polymerisation, even outside of the impact of the Bohr effect, thought to be secondary to the activation of several RBC cationic channels leading to a reduction in intracellular water content and therefore an increase in HbS concentration [77,78]. Further to this, a decrease in blood pH has also been shown to result in an increase in blood viscosity [79]. This will also increase the risk of vaso-occlusive events, independently of the effects of acidosis on HbS polymerisation and RBC sickling. In vitro, studies have shown that acidosis is a strong promoter of HbS polymerisation and therefore RBC sickling. The relative amount of sickled RBCs has been shown to increase markedly from 1% at a pH of 7.4 to >90% at a pH of 7.0 [78]. In vivo studies have also supported that blood acidosis is a strong potential triggering factor for RBC sickling and painful vaso-occlusive crisis [78]. The extent of exercise-induced intramuscular acidosis may be higher in SCD patients, supported by results in mouse models of SCD [80], as well as findings that SCD patients are prone to early lactate accumulation when exercising [59,66]. Taken together, this could suggest that acidotic conditions during exercise could drive HbS sickling and ultimately vaso-occlusive events in patients with SCD.

Increases in temperature that occur during exercise could further decrease HbS affinity to oxygen and also contribute to an increased risk of sickling [17]. Dehydration occurring during exercise could also transiently result in an increased haematocrit and blood viscosity, potentially also promoting vaso-occlusive complications [77]. Increases in adrenaline secondary to acute exercise could also contribute to an increased risk of vaso-occlusion. Adrenaline impacts microvascular blood flow and increases the adhesion of sickle RBCs to the vascular endothelium [81,82].

Results from some studies have suggested that a proportion of SCD patients may be at risk of exercise-induced haemoglobin desaturation, even during mild exercise, such as in a six-minute walking test [83]. The incidence of this amongst paediatric SCD populations has been found to vary from 8% [84] to 42% [85]. This raises concern, as if the haemoglobin desaturation during mild to moderate exercise is significant, it could promote HbS polymerisation and sickling.

Acute exercise also increases the generation of superoxide anions and O_2_-derived intermediates [86]. In addition to damage to the vascular endothelium and increased inflammation, accumulation of reactive oxygen species (ROS) may also directly damage RBCs in SCD patients and further reduce RBC deformability [20].

The impact of acute exercise on blood rheology is another potential concern. Blood rheology is abnormal at baseline in patients with SCD, with a reduction in RBC deformability, increased strength of RBC aggregates, increased plasma viscosity, and increased blood viscosity for a given haematocrit compared to healthy controls [87,88]. Acute exercise increases blood and plasma viscosities, results in a rise in haematocrit, increases RBC aggregation, and decreases RBC deformability, particularly when effort is intense [77,88]. Increased blood viscosity is usually well tolerated in healthy individuals, but this is not the case in SCD patients due to microvascular dysfunction [77,89]. Increased blood viscosity has been found to be an independent risk factor for frequent vaso-occlusive crises in children with SCD [23,77,89]. Thus, the changes seen during acute exercise could increase the risk of vaso-occlusive crisis.

### 3.3. Single Acute Bouts of Exercise May Be Safe and Well-Tolerated

Numerous studies have investigated the impact of single acute bouts of moderate intensity exercise in both children and adults with SCD [90,91,92,93]. Overall, the results of these studies suggest that a single bout of moderate intensity endurance exercise is not associated with major changes in RBC deformability, blood viscosity, inflammation, or oxidative stress. The studies also suggest that this type of exercise is safe in patients with SCD, with no clinical complications or vaso-occlusive crises reported.

Studies involving children and adults with SCD have yielded promising safety data, suggesting that exercising with increasing intensity until exhaustion, such as during maximal cardiopulmonary exercise testing (CPET), appears to be safe and does not result in adverse events [41,73]. In addition, it has been shown that, while baseline levels of inflammatory biomarkers are higher in SCD patients, the magnitude of the acute inflammatory response to maximal CPET is no greater in SCD patients than in healthy controls [41,73]. A systematic review into the safety of maximal CPET in individuals with SCD included data from nearly 1000 children and adults [94]. The review concluded that maximal symptom-limited CPET was safe for individuals with SCD. Less than 4% of patients exhibited any adverse events: 2% showed electrocardiogram (ECG) abnormalities, 0.1% pain requiring hospitalisation, 0.3% pain without hospitalisation, and 1.1% exercise-induced hypoxia. It is likely that CPET is safe in SCD patients, despite its high intensity, due to the relatively short duration, with the incremental phase of testing usually lasting around 10 min in total [95]. It is also only towards the end of the incremental phase that exercise intensity will be higher or maximal, with a gradual ramping up of intensity utilised. CPET protocols also include periods of warming up (the unloaded phase) and cooling down (the recovery phase) that likely contribute to its safety [95]. Studies assessing the impact of higher intensity exercise over longer durations have not been conducted in patients with SCD, likely secondary to fears that it would result in acute complications.

### 3.4. Benefits of Moderate Intensity Aerobic Exercise Training Programmes

In mouse models of sickle cell disease, an eight-week training programme has demonstrated improvements in inflammation, blood viscosity, and oxidative stress [96,97,98,99]. There have been further investigations into the effects of regular exercise for patients with SCD [40,100,101,102,103,104,105,106,107,108,109]. All exercise programmes lasted between 6 and 12 weeks, with two to three exercise sessions per week. A number of studies had participants take part in cycling exercise [40,100,102,103]. Some of these studies targeted an exercise intensity equivalent to a blood lactate concentration of 2.5 mmol/L (the first lactate threshold, LT1) while others targeted an intensity equivalent to either 70% or 100% of the first ventilatory threshold (VT1). Other studies have investigated aerobic exercise programmes involving a combination of walking, calisthenics, and flexibility exercises [101,104,105]. Importantly, the different training sessions were all found to be safe and well-tolerated, with no clinical complications or adverse events reported. A number of beneficial effects have also been identified, including increased aerobic physical fitness and ventilatory efficiency, increased plasma NO, increased muscle capillary density, improved muscle aerobic energetic capacity, and improved scores on standardised measures of quality of life [17,40,100,101,102,103,104,105,106,107,108,109]. It is important, however, to note that the studies had relatively small sample sizes and the various exercise programmes have either been conducted entirely supervised in laboratory settings or been guided by initial supervised laboratory-performed exercise testing, which will limit the generalisability to other settings (Table 1).

A recently conducted study investigated the impact of eight weeks of increased daily step count in SCD patients in Dakar [110]. A key difference from previous studies was that the intervention was simple: an increase in daily step count either by 25% above baseline for 8 weeks, or by 25% above baseline for 4 weeks, and then 50% above baseline for a further 4 weeks. Increasing step count is easy to perform and can be conducted without medical supervision or prior exercise testing. The results of the study suggest that eight weeks of increasing step count by 25–50% is sufficient to increase physical capacity, decrease pain frequency and intensity, improve vascular function, and decrease inflammation in patients with SCD. There were some limitations to the study, most notably that it did not include any women. Further studies are required in different settings where sedentary behaviour is more common and baseline step counts may be lower.

## 4. Regular Exercise, Improved Aerobic Fitness, and the Potential Impact on the Pathophysiology of Sickle Cell Disease

### 4.1. Inflammation

Regular exercise is known to decrease inflammation in healthy individuals [111,112,113,114] and has also been associated with long-term anti-inflammatory effects in various chronic diseases [38,115,116,117,118]. Exercise may have anti-inflammatory effects through the release of IL-6 from contracting skeletal muscle [119]. Acting as a myokine, IL-6 is thought to drive increased levels of the anti-inflammatory cytokines IL-10 and IL-1 receptor antagonist (IL-1ra), as well as decreased levels of TNF-α [38,117]. In a mouse model of SCD, it has been shown that eight weeks of aerobic training resulted in decreased levels of several markers of systemic inflammation, including white blood cell count and IL-1β [97], suggesting that regular physical activity in SCD may result in reduced inflammation. Indeed, decreased IL-6 and TNF-α levels have been found in SCD patients following a 12-week exercise programme [106], and decreased IL-6, TNF-α, and IFN-γ levels were found in SCD patients who had increased their step count for an eight-week period when compared to a control group, although there was some variation in results between the study’s two different intervention groups [110].

Given that elevation of IL-6 in the steady state is associated with an impaired immune response and increased morbidity [75], long-term decreases in IL-6 secondary to regular exercise could be of clinical benefit in patients with SCD. Indeed, studies have suggested that, in general, a profile of low IL-10 but high IL-6 and TNFα is seen in patients who have more frequent vaso-occlusive crises, whereas the inverse (high IL-10, lower IL-6 and TNFα) is seen in patients who have not had a vaso-occlusive crisis in the last nine months [120]. Other studies have provided further support for the benefit of a low IL-6 and high IL-10 environment. A study in adult SCD patients has shown negative correlations between IL-6 values and both the distance walked on a treadmill and estimated peak VO2 values [104]. Reduced levels of IL-10 have been found to be correlated with the frequency, severity, and duration of vaso-occlusive crises in SCD patients [74]. Regular physical activity has been shown to result in markedly increased IL-10 levels in the context of other chronic diseases, such as type 2 diabetes mellitus (following a six-month exercise training programme) [117,121] as well as in post-myocardial infarction patients (following an eight-week exercise training programme) [122]. If regular exercise can result in a favourable shift in these inflammatory mediators in patients with SCD, driving a decrease in IL-6 and an increase in IL-10, it may reduce the frequency of vaso-occlusive events and, ultimately, the clinical burden of the disease.

Increased levels of anti-inflammatory cytokines driven by exercise are also suspected to drive downregulation of TLR4 expression on innate immune cells [116,123]. In healthy individuals of different ages, a 12-week exercise programme has been shown to result in a significant reduction in TLR4 expression [124]. Reduced TLR4 expression may well have a clinical benefit: TLR4-mediated signalling is felt to drive inflammation and vasculopathy in response to haemolytic products released into the blood [11,17]. In mouse models of SCD, pharmacological inhibition or genetic knockdown of TLR4 has demonstrated protective effects against the development of ACS following heme administration [125,126,127]. Decreased expression of TLR4 following regular physical exercise could well attenuate this element of SCD pathophysiology and contribute to reduced inflammation and reduced vasculopathy.

Visceral fat loss secondary to regular physical activity also plays a role in the anti-inflammatory effect seen. Visceral adipose tissue is known to produce a number of inflammatory adipokines, including TNF-α, IL-6, and IL-18 [117]. Excessive visceral adipose tissue is also thought to result in a reduction in plasma anti-inflammatory mediators, together contributing to a chronic low-grade inflammatory state [116,117].

### 4.2. Vascular Dysfunction, Endothelial Activation, and Cell Adhesion

Regular physical activity has also been shown to have beneficial effects on endothelial activation. Cross-sectional studies have shown that levels of physical activity negatively correlate with endothelial activation in healthy individuals [128,129]. Further, ageing-related increases in plasma sICAM-1 have been shown to be attenuated by regular physical activity [130]. In patients with other chronic conditions such as heart failure, peripheral arterial disease, or type 2 diabetes mellitus, regular physical activity has been shown to result in lower levels of sVCAM-1 [118], sICAM-1 [131,132], sP-selectin [131,133], and sE-selectin [132]. This process may be mediated by the rise in IL-10 seen following regular exercise [117,121,122], with IL-10 known to downregulate the expression of both VCAM-1 and ICAM-1 [134].

Studies with mouse models of SCD, despite limitations secondary to the translational uncertainty of their results to human disease and human subjects, have also supported a role for regular physical activity in reducing endothelial activation and dysfunction. Sedentary sickle mice exposed to hypoxia/reoxygenation stress show increased levels of pulmonary VCAM-1 [135]. In comparison, sickle mice that had undergone eight weeks of voluntary wheel running did not show this same increase in pulmonary VCAM-1 levels [136]. This suggests that regular physical activity can reduce endothelial activation and improve vascular function in SCD mice.

Human studies have so far mostly focused on acute bouts of exercise rather than regular exercise training and have yielded differing results. A study in the Ivory Coast measured markers of endothelial activation in 11 SCD patients following 20 min of cycling exercise and found a slight increase in sVCAM-1 in both SCD and control groups, and a slight increase in sICAM-1 in just the SCD group [92]. In contrast, a study in the US in 90 children and adolescents who underwent maximal exercise testing found that SCA patients had a higher level of sVCAM-1 at baseline compared to healthy controls, but that the exercise did not induce any further rise [73]. A lower fitness level, defined by peak oxygen consumption (VO2), was independently associated with a greater acute phase response to exercise for sVCAM-1 across SCA patients and healthy controls.

A recent cross-sectional study of 98 adults with SCD in Senegal found an association between higher daily step count and improved vascular function in adults with SCD [137]. Vascular function was assessed using carotid-femoral and carotid-radial pulse wave velocity (CF-PWV and CR-PWV) as a proxy measure for arterial stiffness. A recent interventional study investigating the impact of eight weeks of increased step count in SCD patients looked at vascular function as an endpoint [110]. CF-PWV and CR-PWV decreased in both intervention groups, and there was a significant negative correlation between changes in six-minute walking distance and decreased CF-PWV. In addition, the percentage of HUVECs (human umbilical vein endothelial cells) positive for ICAM-1 after incubation with patient plasma was significantly decreased in the second intervention group, suggesting reduced endothelial activation.

Further in vivo studies exploring the impact of longer-term regular physical activity on endothelial activation and the expression of these adhesion molecules in SCD patients are required to see whether the promising results seen in other populations with different chronic diseases can be replicated.

### 4.3. Blood Rheology

Regular physical activity in the general population has been found to result in a number of changes in blood rheology, including an improvement in RBC deformability [138,139,140] and a decrease in blood viscosity [140,141,142,143]. A study in a mouse model of SCD has shown that mice that undertook regular physical activity had a decrease in blood viscosity compared to their sedentary counterparts [98]. Human studies on the impact of exercise on blood rheology in sickle cell patients have so far focused on single acute bouts of exercise. One study found blood viscosity and haematocrit were unchanged compared to resting levels in SCD patients following a 20 min symptom-limited exercise test on an ergocycle, although a mild increase in the percentage of dense sickled RBCs was seen [91]. Another study has demonstrated there was no further alteration in RBC deformability from baseline in SCD patients following 10–12 min of submaximal acute exercise on an ergocycle [90]. The same study also subsequently found that the strength of RBC aggregates was decreased in samples taken two and three days after exercise. This may well be of clinical benefit: the strength of RBC aggregates has been associated with the risk of acute chest syndrome (ACS) [23]. A recent cross-sectional study of adults with SCD in Senegal, cited above for its findings regarding vascular function, has also found an association between higher daily step counts and reduced blood viscosity [137]. Future interventional studies should be conducted to explore the impact of regular exercise training programmes on blood rheology in SCD patients.

### 4.4. NO Metabolism and Oxidative Stress

Regular physical activity, over a period of weeks or months, has been shown to result in upregulation of endothelial NO bioactivity [144] and increased NO bioavailability [145]. Studies have also shown that RBCs themselves are able to produce NO [146], as well as that regular physical activity increases the activity of RBC endothelial-like NO synthase (NOS), resulting in an improvement in vascular reactivity [139]. Other potentially beneficial effects resulting from regular physical activity, seen in healthy populations and in individuals with cardiac disease, include a decreased expression of ROS-forming enzymes and enhanced endogenous antioxidant defences, such as upregulation of the activity of RBC catalase and glutathione reductase [117,145,147,148]. A favourable shift in inflammatory profile following regular exercise, including a rise in IL-10, may also play a role in the benefits seen, with IL-10 having been demonstrated to suppress the production of ROS [149,150].

Studies conducted with mouse models of SCD have shown promising results with regard to the impact of regular exercise on NO metabolism. Eight weeks of voluntary exercise training has been shown to decrease oxidative stress, with increased NO production, endothelial NOS (eNOS) mRNA expression, and eNOS activation all found to be increased in the lungs of exercised mice compared to sedentary controls following hypoxia/reoxygenation stress [96].

Studies conducted in the human SCD population have focused on acute bouts of exercise. Comparison of SCD patients and healthy controls who performed an acute submaximal exercise test found that the RBC NO level was higher in SCA patients at rest and decreased significantly after exercise, and that free radical levels, while higher in SCA patients at rest, were not affected by exercise [93]. Markers of oxidative stress in 11 SCA patients following an acute bout of moderate endurance exercise (20 min of cycling) were found to be unchanged [92]. Thirty minutes of moderate intensity exercise conducted over three consecutive days in SCD patients has also been found to result in a rise in plasma NO [151]. A study investigating the impact of a six-week exercise training programme involving cycling in SCD patients found evidence for reduced nitrosative stress and improved NO bioavailability following the exercise training [40]. The same study also found that plasma free haemoglobin concentration was reduced following the exercise programme, with plasma free haemoglobin known to play an important role in the pathophysiology of SCD through generating ROS as well as scavenging NO and creating a functional NO deficiency [9,10,11]. Overall, the results of these studies suggest that acute exercise does not result in a deleterious increase in oxidative stress in SCD patients. Further studies are required to confirm the effects of longer-term regular physical activity on NO metabolism and oxidative stress.

### 4.5. Cardiac Effects

Regular physical activity is beneficial for cardiovascular health [152]. Studies have shown that exercise-trained individuals have improved systolic and diastolic function, including a faster diastolic filling rate and a faster left ventricular emptying rate [153]. Beneficial effects on cardiac function have also been demonstrated in SCD patients. A study on the impact of an eight-week exercise programme in 27 adults with SCD demonstrated significant improvement in cardiovascular results following the exercise programme, including an increased ejection fraction and an improvement in diastolic function [101]. Another study investigating the impact of an eight-week exercise programme in SCD patients also demonstrated an improvement in systolic function in the exercise group, with an improved ejection fraction [104]. The authors suggested that this may be related to a reduction in afterload secondary to improved endothelial function, with a similar mechanism having been suggested to be responsible for similar benefits seen in patients with heart failure [154]. Increased NO production may underlie this process and has been seen in studies investigating the effects of regular physical activity in patients with heart failure [155]. Providing further support for this as a potential mechanism of improvement in endothelial function following exercise, increased NO levels have been seen in SCD patients following participation in moderate-intensity exercise protocols [151].

Diastolic dysfunction has been shown to be an independent risk factor for death in SCD patients [156]. If regular exercise can lead to an improvement in cardiac function, this could improve health and decrease mortality.

### 4.6. Respiratory Function

Regular exercise training has been shown to result in improvements in pulmonary function in patients with chronic respiratory diseases, such as improved gas exchange, decreased respiratory symptoms, and improved functional capacity [157,158]. A large cohort study of UK Biobank participants found that higher levels of physical activity are associated with a slower decline in lung function with age in the general population [159]. Given that lung function abnormalities are frequently seen in children and adolescents with SCD and decline with increasing age [53,54], such a slowing in decline could be very beneficial.

There are relatively few studies investigating the impact of regular exercise on the pulmonary function in SCD patients. Regular exercise training for six to eight weeks in SCD patients has been found to result in an increase in ventilatory efficiency [40], as well as in a lower respiratory frequency for a given submaximal power output, suggesting improved ventilatory function [63]. A significant improvement in maximal inspiratory pressure (MIP) and maximal expiratory pressure (MEP) has also been found following a 12-week exercise programme in SCD patients [105]. MIP and MEP have been found to be below predicted values in patients with SCD at baseline [160].

### 4.7. Muscle Structure and Function

In healthy individuals, regular endurance exercise training results in improved type I muscle fibre cross-sectional area and improved oxidative enzyme activities [63], as well as microvascular benefits [100]. Endurance training has been shown to reduce exercise-induced intramuscular acidosis and improve muscle function in a mouse model of SCD [161]. Markers of oxidative stress in muscle tissue have also been shown to be reduced following exercise training in sickle mice [99,161].

In human studies, an eight-week exercise programme in SCD patients resulted in a gain of skeletal muscle microvasculature [100], as well as increased type I muscle fibre surface area and increased oxidative enzyme activity [63]. Alongside these histological changes, improved exercise capacity, lower blood lactate concentration, and lower ratings of perceived exertion were also seen in the patients who underwent the training programme. A significant improvement in both handgrip and quadricep strength following a 12-week exercise training programme has also been demonstrated in SCD patients [105]. These results suggest that moderate intensity regular exercise can partially reverse the microvascular and muscular deficits commonly observed in the skeletal muscle of SCD patients, with a corresponding functional improvement also suggested.

### 4.8. Impact on Clinical Symptoms

The results obtained from various studies investigating the impact of regular exercise programmes in SCD patients are encouraging, but evidence tying the potential improvements seen in various underlying factors, such as inflammation or vascular function, to improvements in clinical condition is more limited at present.

Some studies have suggested that improved conditioning and physical fitness may result in an improvement in the quality of life of patients with SCD. Following exercise training, SCD patients had a lower rating of perceived exertion for a given submaximal power output [63] and had significant improvements in functional capacity, especially for exercise levels close to those needed for daily activities [103]. The daily activity level of the children and adolescents has been measured both before a period of exercise training and one year after, and has been found to be increased in 80% of cases [40]. Improved physical fitness may improve quality of life by making it easier for patients with SCD to comfortably perform activities of daily living requiring mild to moderate physical activity. A cross-sectional study conducted in Senegal has found higher daily step counts to be correlated with lower pain frequency and intensity in SCD patients, but not with the frequency of vaso-occlusive crises [137,162]. In addition, the frequency of pain, mean pain intensity, and the degree of interference with daily activities, when assessed using a standardised questionnaire, have all been found to decrease following eight weeks of increased daily step count in SCD patients [110]. Other studies involving eight-week exercise training programmes in SCD patients have shown significant improvements in the scores on standardised quality of life metrics (the 36-Item Short Form Survey, SF-36) in the groups that undertook the exercise programme [104,105].

Future studies should focus on whether regular exercise training results in associated measurable clinical improvement for SCD patients. More studies are required to demonstrate an impact on pain intensity and frequency, as well as on quality of life. No longitudinal or interventional studies to date have investigated whether regular exercise results in a decrease in the frequency of vaso-occlusive crises.

## 5. Exercise Recommendations in SCD

Clear generalised guidelines for exercise in the SCD population would be of benefit for patients, their families, and clinicians. Such guidelines do not yet exist. Further studies are needed to guide the development of a set of evidence-based guidelines, ensuring recommendations are safe and widely applicable. Such future guidelines should detail how much exercise is safe, what types of exercise are safe, and what precautions should be taken around exercise.

Guidance on the intensity and duration of exercise that can be conducted safely will be a key part of any generalised exercise guidelines. Further work is required to identify the exercise intensity that provides the maximum benefit for the minimum risk in the SCD population. There is a dose–response relationship between exercise and health outcomes, with physical activity above the minimum recommended amounts resulting in more significant improvements, but extremely high-intensity exercise, or exercise without appropriate resting periods, is associated with adverse events [72,117]. Many of the studies conducted investigating regular exercise training in SCD patients to date have used baseline exercise testing, such as CPET, to help determine a safe level of exercise. In lower resource settings in particular, access to formal exercise testing is likely to be much more limited. There are sizeable discrepancies between outcomes in high-resource and low-resource settings for patients with SCD. For example, 99% of children with SCD in London survive to adulthood [163], compared to less than 50% in Tanzania [164]. Differences in access to various treatments, such as antibiotic prophylaxis, hydroxycarbamide, or blood transfusions, are a key factor in this health inequity [1,165]. Regular exercise has no or at least limited cost and is an intervention that is more easily generalisable across different populations in both low and high-resource settings. Generalised guidelines should therefore detail forms and intensities of exercise that can be safely practised in situations where formal exercise testing cannot be conducted. In the absence of specific CPET data, individual differences in disease severities, as well as any individual history of exercise-related symptoms or adverse events, should be taken into account when determining what intensity of exercise can be safely targeted. Simpler tests, such as a six-minute walking test, can also be utilised to obtain some general information on exercise capacity. CPET and resulting personalised exercise calibration can still play a role where it is available, and the extra information obtained may allow for relatively higher intensities of exercise to be deemed safe, particularly in patients with more severe disease and lower levels of physical fitness, where extra caution will be required. The potential impact of other significant co-morbidities that would also likely affect exercise tolerance, such as asthma (for which SCD has been found to be a risk factor, independent of familial and environmental factors [166]), will be another key consideration during the development of future guidelines.

When seeking to determine the intensity of exercise that will be safe for patients with different clinical profiles to perform, it is possible that other measurable metrics, such as foetal haemoglobin (HbF) concentration, could play a role. HbF concentration is inherited as a quantitative genetic trait, with a number of major loci identified [167]. Higher HbF levels reduce intracellular HbS concentrations and interrupt polymerisation, and there is evidence that the maintenance of high foetal haemoglobin (HbF) concentrations after infancy results in a number of clinical benefits, including increased life expectancy [24] and reduced frequency of acute pain [27]. It is possible that higher HbF concentrations might also be beneficial for a patient’s exercise tolerance, but studies assessing the impact of different HbF concentrations on exercise tolerance are limited to date. One study of 42 children with SCD did find a significant correlation between HbF levels and performance in a six-minute walk test [168]. Conversely, another recent study assessing the response of 38 adults with SCD to an eight-week period of increased daily step count found no association between the benefits seen (including increased distance walked in the six-minute walk test, reduced pain intensity and frequency, some decreased inflammatory markers, and improved vascular function) and HbF levels [110]. Further studies with larger sample sizes to confirm and quantify any potential benefits of higher HbF concentrations on exercise tolerance are required. The co-inheritance of α-thalassaemia is also thought to modify the phenotype of SCD. Co-inheritance of α-thalassaemia reduces the concentration of haemoglobin in each erythrocyte and decreases the tendency of HbS to polymerise. Increased haemoglobin concentrations and decreased rates of haemolysis are seen in these patients [3]. Clinical effects are variable: for example, co-inheritance of α-thalassaemia has been shown to reduce the incidence of cerebral vasculopathy [169,170,171], but conversely does not reduce pain frequency or may even increase it [172]. There is no increase in survival [173]. The impact of co-inherited α-thalassaemia on exercise tolerance in SCD patients is unknown, and exploring this in future studies would also be of benefit.

Generalised guidelines will also need to advise on what forms of exercise are safe and can be recommended to SCD patients. Based on the studies conducted so far, it appears that moderate intensity cycling, walking, calisthenic exercises, and resistance training can be practised safely [17]. The potential safety of team sports is another consideration. Team sports increase the opportunity for social interactions, and studies have shown that there are particular mental health benefits seen following participation in organised team sports that are either not as pronounced or not seen at all with participation in individually performed exercise [174,175]. Potential benefits need to be weighed against potential risks, such as the risk of abdominal impacts in contact sports and the potential rupture of an enlarged spleen [17]. A pilot study in the Netherlands had eight children with SCD between the ages of 7 and 12 participate in a 10-week programme involving playing football (soccer) and other football-related activities for 90 min, and found this form of moderate-to-vigorous intensity organised team sport was safe, with no adverse events or vaso-occlusive crises related to the exercise occurring [176]. Regular water breaks and additional short breaks as required were implemented throughout. Importantly, it also appears that the children in the study enjoyed themselves. At baseline, none of them participated in any organised sports activities. Following the conclusion of the study, every single participant either continued participating in the same training programme or an alternative similar organised sports activity. This will not be the case for all potential training programmes. A study of a home exercise programme in the US found decreased adherence to their exercise programme in the second part of their study, with some of the adolescents involved expressing decreased interest and motivation [102]. Continued motivation and adherence to an exercise programme over a longer period will be vital to ensure continued health benefits and is a factor that future guidelines should consider.

Advice should also be given on precautions to take during exercise that are likely to be important for SCD patients, such as frequent hydration, which will help limit rises in blood viscosity and core body temperature during exercise [17,77,177]. Appropriate periods of warming up and cooling down, either side of exercise, are also likely to be important in the SCD population [17], and this should similarly be reflected in the guidance provided.

Guidelines should also consider the impact of various environmental factors on the safety of exercise in the SCD population. Training programmes studied so far have largely been conducted either in the laboratory or at home, and it is not known to what extent climatic factors may influence safety. For example, cold temperatures are commonly reported by patients to be a precipitant of acute pain [178], and hot temperatures may lead to an increased risk of dehydration and cardiac strain [17]. Further studies are required to determine the degree to which different temperatures might impact exercise safety, as well as the degree to which any risks can be alleviated by sensible precautions being taken, such as the use of appropriate clothing and appropriate hydration strategies. Exercise conducted outside in urban areas may also result in increased exposure to air pollution, which has been linked to adverse effects, including increased hospital visits and abnormal pulmonary function in SCD patients [179,180,181]. The majority of the published data appears to support a beneficial effect of exercise even at higher air pollution levels, such as levels of fine particulate matter with a diameter less than 2.5 micrometres (PM2.5) or levels of coarse particulate matter with a diameter less than 10 micrometres (PM10) above the 70th percentile for exposure in a study in South Korea (PM2.5 > 27.86 μg/m^3^ and PM10 > 55.13 μg/m^3^) [182] or above the median concentration seen across a five-year period in another study in Sweden, where average pollution levels are relatively lower (PM2.5 > 5.7 μg/m^3^ and PM10 > 9.6 μg/m^3^) [183,184]. Further research into this area is needed, with a particular focus on investigating the effects of exercising in polluted air in both children and patients with SCD.

## 6. Future Directions

When looking forward to potential future research work to be done, it is important to consider the key limitations of the studies to date that limit the strength of the conclusions we can draw from them. For example, most have small sample sizes. Further, while inclusion criteria vary from study to study, the enrolled SCD patients tend to be relatively healthy and have, for example, not had a recent vaso-occlusive crisis. Future studies with larger cohorts would be valuable, as would studies cautiously investigating the impact and potential benefits of exercise for patients with more severe clinical profiles. It is also important to consider that the training programmes undertaken remain relatively short, with studies conducted to date having exercise programmes of between 6 and 12 weeks. Future work to explore the impact of longer-term regular exercise would also be of great value to assess both the long-term safety of such an intervention in this population, as well as whether the potential benefits seen are sustained or indeed further improved. Some studies are also limited by the exercise taking place in highly controlled laboratory settings. Another area of future focus should be on evaluating the impact of exercise regimens that are easy to implement and can be widely introduced, including in lower-resource settings, where detailed pre-training exercise testing is unlikely to be feasible.

Studies may yield promising results from signs of reduced inflammation to improved muscle microvasculature on biopsy, but these physiological improvements do not guarantee that an accompanying clinical benefit will be seen. Relatively few studies to date have assessed clinically meaningful outcomes following regular exercise training in SCD patients. Future studies should investigate whether regular exercise training in SCD patients results in clinically meaningful benefits, such as decreased pain or a decreased frequency of presentations to hospital with vaso-occlusive events.

A key overall focus of future work should be to identify an optimal level of exercise that results in maximum benefit with minimum risk and to develop evidence-based guidelines for exercise for the SCD population.

## 7. Conclusions

Regular exercise training can potentially positively impact pathophysiological processes underlying sickling and vaso-occlusion in patients with SCD. In addition, it can result in improvements in cardiopulmonary and muscular function and potentially in quality of life. Importantly, and in contrast to other potential interventions, regular exercise has no or at least relatively limited cost and is easily generalisable across different populations.

## Figures and Tables

**Table 1:** Studies assessing the impact of 6-to-12-week exercise training programmes in patients with SCD.

Author + Year	Number of Participants	Type of Exercise	Duration of Training Programme	Intensity Targeted	Beneficial Outcomes Reported
Grau et al. 2019 [40]	12 children and young adults with SCD	Two sessions per week of 15–30 min of cycling exercise	6 weeks	70% of the first ventilatory threshold (VT1) (based on pre-training CPET)	Improved ventilatory efficiency Reduced nitrosative stress, reduced plasma free Hb concentration, and increased plasma nitrite levels Increase in daily life activity level scored by questionnaire
Merlet et al. 2019 [100] and 2020 [63]	40 adults with SCD	Three sessions per week of 40 min of cycling exercise	8 weeks	Power output corresponding to 2.5 mmol/L blood lactate concentration (LT1) (based on pre-training CPET)	Improved skeletal muscle microvasculature Increased type I muscle fibre surface area and increased muscle oxidative enzyme activity Lower respiratory frequency, lower blood lactate concentration, and lower rating of perceived exertion at a given power output
De Araujo Junior et al. 2021 [101]	27 adults with SCD	Three to five sessions per week of 45–60 min of home-based aerobic exercises, including walking, calisthenics, and flexibility exercises	8 weeks	60–75% of HRmax (based on pre-training treadmill test)	Increase in distance travelled on treadmill test Increased ejection fraction Improved diastolic function
Liem et al. 2017 [102]	10 children with SCD	Three sessions per week of 30 min of cycling exercise	12 weeks	100% of the first ventilatory threshold (VT1) (based on pre-training CPET)	Improved mean peak VO2 and peak workload after 6 weeks No increase in inflammatory markers
Gellen et al. 2018 [103]	40 adults with SCD	Three sessions per week of 45 min of cycling exercise	8 weeks	Power output corresponding to 2.5 mmol/L blood lactate concentration (LT1) (based on pre-training CPET)	Improved lactate thresholds during exercise Increased capillary density on muscle biopsy
Antonelli Rossi et al. 2023 [104]	53 adults with SCD	Three sessions per week of 60 min of home-based aerobic exercises, including walking, calisthenics, and flexibility exercises	8 weeks	60–75% of HRmax (based on pre-training treadmill test)	No increase in inflammatory markers Increase in distance travelled on treadmill Improvement in peak VO2 Improved right ventricular systolic function and left ventricular ejection fraction Improvement in scores on standardised quality of life questionnaire (SF-36)
Almeida et al. 2021 [105]	40 adults with SCD	Three sessions per week of 60 min of muscle training, aerobic resistance, and flexibility exercises	12 weeks	Intensity guided by a physical therapist	Increased six-minute walking distance Increases in maximal inspiratory pressure (MIP) and maximal expiratory pressure (MEP) Increase in handgrip and quadriceps strength Improvement in scores on standardised quality of life questionnaire (SF-36)
El-Kader and Al-Shreef 2018 [106]	60 adults with SCD	Three sessions per week of 30 min of treadmill exercise	12 weeks	60–70% of HRmax	Decrease in inflammatory markers in the exercise group, including TNF-α and IL-6
De Lima et al. 2026 [110]	38 adults with SCD	Increases in daily step count by either 25% or 50%	8 weeks	N/a (Increase in daily step count rather than specific exercise sessions)	Increased six-minute walking distance Decreased pain intensity and frequency Decreased arterial stiffness Decreases in some inflammatory markers and in markers of endothelial activation

## Data Availability

No new data were created or analysed in this study.

## Abbreviations

The following abbreviations are used in this manuscript:

SCD	Sickle cell disease
HbS	Sickle haemoglobin
VCAM-1	Vascular cell adhesion molecule-1
ICAM-1	Intracellular adhesion molecule-1
ROS	Reactive oxygen species
NO	Nitric oxide
RBC	Red blood cell
ACS	Acute chest syndrome
TLR4	Toll-like receptor 4
HbF	Foetal haemoglobin
VO2	Oxygen consumption
CPET	Cardiopulmonary exercise testing
CF-PWV	Carotid-femoral pulse wave velocity
CR-PWV	Carotid-radial pulse wave velocity
NOS	Nitric oxide synthase
eNOS	Endothelial nitric oxide synthase
MIP	Maximal inspiratory pressure
MEP	Maximal expiratory pressure
PM	Particulate matter

## References

[1] Rees D.C., Brousse V.A.M., Brewin J.N. (2022). Determinants of Severity in Sickle Cell Disease. Blood Rev..

[2] Wastnedge E., Waters D., Patel S., Morrison K., Goh M.Y., Adeloye D., Rudan I. (2018). The Global Burden of Sickle Cell Disease in Children under Five Years of Age: A Systematic Review and Meta-Analysis. J. Glob. Health.

[3] Rees D.C., Williams T.N., Gladwin M.T. (2010). Sickle-Cell Disease. Lancet.

[4] Connes P., Alexy T., Detterich J., Romana M., Hardy-Dessources M.-D., Ballas S.K. (2016). The Role of Blood Rheology in Sickle Cell Disease. Blood Rev..

[5] Belcher J.D., Mahaseth H., Welch T.E., Vilback A.E., Sonbol K.M., Kalambur V.S., Bowlin P.R., Bischof J.C., Hebbel R.P., Vercellotti G.M. (2005). Critical Role of Endothelial Cell Activation in Hypoxia-Induced Vasoocclusion in Transgenic Sickle Mice. Am. J. Physiol.-Heart Circ. Physiol..

[6] Wung B.S., Ni C.W., Wang D.L. (2005). ICAM-1 Induction by TNFalpha and IL-6 Is Mediated by Distinct Pathways via Rac in Endothelial Cells. J. Biomed. Sci..

[7] Gupta P., Kumar R. (2024). Targeting ICAM1 to Ameliorate Vaso-Occlusion and Inflammation in Sickle Cell Disease. Eur. J. Haematol..

[8] Pierrot-Gallo B.S., Vicari P., Matsuda S.S., Adegoke S.A., Mecabo G., Figueiredo M.S. (2015). Haptoglobin Gene Polymorphisms and Interleukin-6 and -8 Levels in Patients with Sickle Cell Anemia. Rev. Bras. Hematol. E Hemoter..

[9] Repka T., Hebbel R.P. (1991). Hydroxyl Radical Formation by Sickle Erythrocyte Membranes: Role of Pathologic Iron Deposits and Cytoplasmic Reducing Agents. Blood.

[10] Reiter C.D., Wang X., Tanus-Santos J.E., Hogg N., Cannon R.O., Schechter A.N., Gladwin M.T. (2002). Cell-Free Hemoglobin Limits Nitric Oxide Bioavailability in Sickle-Cell Disease. Nat. Med..

[11] Nader E., Romana M., Connes P. (2020). The Red Blood Cell—Inflammation Vicious Circle in Sickle Cell Disease. Front. Immunol..

[12] De Caterina R., Libby P., Peng H.B., Thannickal V.J., Rajavashisth T.B., Gimbrone M.A., Shin W.S., Liao J.K. (1995). Nitric Oxide Decreases Cytokine-Induced Endothelial Activation. Nitric Oxide Selectively Reduces Endothelial Expression of Adhesion Molecules and Proinflammatory Cytokines. J. Clin. Investig..

[13] Palmer R.M.J., Ashton D.S., Moncada S. (1988). Vascular Endothelial Cells Synthesize Nitric Oxide from L-Arginine. Nature.

[14] Morris C.R., Kato G.J., Poljakovic M., Wang X., Blackwelder W.C., Sanchdev V., Hazen S.L., Vichinsky E.P., Morris S.M., Gladwin M.T. (2005). Dysregulated Arginine Metabolism, Hemolysis-Associated Pulmonary Hypertension and Mortality in Sickle Cell Disease. JAMA J. Am. Med. Assoc..

[15] Möckesch B., Connes P., Charlot K., Skinner S., Hardy-Dessources M.-D., Romana M., Jumet S., Petras M., Divialle-Doumdo L., Martin C. (2017). Association between Oxidative Stress and Vascular Reactivity in Children with Sickle Cell Anaemia and Sickle Haemoglobin C Disease. Br. J. Haematol..

[16] Adisa O.A., Hu Y., Ghosh S., Aryee D., Osunkwo I., Ofori-Acquah S.F. (2013). Association between Plasma Free Haem and Incidence of Vaso-Occlusive Episodes and Acute Chest Syndrome in Children with Sickle Cell Disease. Br. J. Haematol..

[17] Connes P., Stauffer E., Liem R.I., Nader E. (2024). Exercise and Training in Sickle Cell Disease: Safety, Potential Benefits, and Recommendations. Am. J. Hematol..

[18] Connes P., Lamarre Y., Waltz X., Ballas S.K., Lemonne N., Etienne-Julan M., Hue O., Hardy-Dessources M.-D., Romana M. (2014). Haemolysis and Abnormal Haemorheology in Sickle Cell Anaemia. Br. J. Haematol..

[19] Baskurt O.K., Temiz A., Meiselman H.J. (1998). Effect of Superoxide Anions on Red Blood Cell Rheologic Properties. Free Radic. Biol. Med..

[20] Hierso R., Waltz X., Mora P., Romana M., Lemonne N., Connes P., Hardy-Dessources M.-D. (2014). Effects of Oxidative Stress on Red Blood Cell Rheology in Sickle Cell Patients. Br. J. Haematol..

[21] Cabrales P. (2007). Effects of Erythrocyte Flexibility on Microvascular Perfusion and Oxygenation during Acute Anemia. Am. J. Physiol.-Heart Circ. Physiol..

[22] Parthasarathi K., Lipowsky H.H. (1999). Capillary Recruitment in Response to Tissue Hypoxia and Its Dependence on Red Blood Cell Deformability. Am. J. Physiol.-Heart Circ. Physiol..

[23] Lamarre Y., Romana M., Waltz X., Lalanne-Mistrih M.-L., Tressières B., Divialle-Doumdo L., Hardy-Dessources M.-D., Vent-Schmidt J., Petras M., Broquere C. (2012). Hemorheological Risk Factors of Acute Chest Syndrome and Painful Vaso-Occlusive Crisis in Children with Sickle Cell Disease. Haematologica.

[24] Platt O.S., Brambilla D.J., Rosse W.F., Milner P.F., Castro O., Steinberg M.H., Klug P.P. (1994). Mortality in Sickle Cell Disease—Life Expectancy and Risk Factors for Early Death. N. Engl. J. Med..

[25] Osunkwo I., Andemariam B., Minniti C.P., Inusa B.P.D., El Rassi F., Francis-Gibson B., Nero A., Trimnell C., Abboud M.R., Arlet J. (2021). Impact of Sickle Cell Disease on Patients’ Daily Lives, Symptoms Reported, and Disease Management Strategies: Results from the International Sickle Cell World Assessment Survey (SWAY). Am. J. Hematol..

[26] McClish D.K., Penberthy L.T., Bovbjerg V.E., Roberts J.D., Aisiku I.P., Levenson J.L., Roseff S.D., Smith W.R. (2005). Health Related Quality of Life in Sickle Cell Patients: The PiSCES Project. Health Qual. Life Outcomes.

[27] Platt O.S., Thorington B.D., Brambilla D.J., Milner P.F., Rosse W.F., Vichinsky E., Kinney T.R. (1991). Pain in Sickle Cell Disease. N. Engl. J. Med..

[28] Bernaudin F., Verlhac S., Arnaud C., Kamdem A., Chevret S., Hau I., Coïc L., Leveillé E., Lemarchand E., Lesprit E. (2011). Impact of Early Transcranial Doppler Screening and Intensive Therapy on Cerebral Vasculopathy Outcome in a Newborn Sickle Cell Anemia Cohort. Blood.

[29] Brousse V., Makani J., Rees D.C. (2014). Management of Sickle Cell Disease in the Community. BMJ.

[30] Heitzer A.M., Hamilton L., Stafford C., Gossett J., Ouellette L., Trpchevska A., King A.A., Kang G., Hankins J.S. (2021). Academic Performance of Children with Sickle Cell Disease in the United States: A Meta-Analysis. Front. Neurol..

[31] Gordon R.D., Welkie R.L., Quaye N., Hankins J.S., Kassim A.A., Thompson A.A., Treadwell M., Lin C.J., Cronin R.M. (2024). Burden of Employment Loss and Absenteeism in Adults and Caregivers of Children with Sickle Cell Disease. Blood Adv..

[32] Dhuli K., Naureen Z., Medori M.C., Fioretti F., Caruso P., Perrone M.A., Nodari S., Manganotti P., Xhufi S., Bushati M. (2022). Physical Activity for Health. J. Prev. Med. Hyg..

[33] Ruegsegger G.N., Booth F.W. (2018). Health Benefits of Exercise. Cold Spring Harb. Perspect. Med..

[34] Banach M., Lewek J., Surma S., Penson P.E., Sahebkar A., Martin S.S., Bajraktari G., Henein M.Y., Reiner Ž., Bielecka-Dąbrowa A. (2023). The Association between Daily Step Count and All-Cause and Cardiovascular Mortality: A Meta-Analysis. Eur. J. Prev. Cardiol..

[35] Blair S.N., Kampert J.B., Kohl H.W., Barlow C.E., Macera C.A., Paffenbarger R.S., Gibbons L.W. (1996). Influences of Cardiorespiratory Fitness and Other Precursors on Cardiovascular Disease and All-Cause Mortality in Men and Women. JAMA.

[36] Blair S.N., Kohl H.W., Barlow C.E., Paffenbarger R.S., Gibbons L.W., Macera C.A. (1995). Changes in Physical Fitness and All-Cause Mortality: A Prospective Study of Healthy and Unhealthy Men. JAMA.

[37] Booth F.W., Roberts C.K., Laye M.J. (2012). Lack of Exercise Is a Major Cause of Chronic Diseases. Compr. Physiol..

[38] Mathur N., Pedersen B.K. (2008). Exercise as a Mean to Control Low-Grade Systemic Inflammation. Mediat. Inflamm..

[39] Olorunyomi O.O., Liem R.I., Hsu L.L. (2022). Motivators and Barriers to Physical Activity among Youth with Sickle Cell Disease: Brief Review. Children.

[40] Grau M., Nader E., Jerke M., Schenk A., Renoux C., Dietz T., Collins B., Bizjak D.A., Joly P., Bloch W. (2019). Impact of A Six Week Training Program on Ventilatory Efficiency, Red Blood Cell Rheological Parameters and Red Blood Cell Nitric Oxide Signaling in Young Sickle Cell Anemia Patients: A Pilot Study. J. Clin. Med..

[41] Liem R.I. (2018). Balancing Exercise Risk and Benefits: Lessons Learned from Sickle Cell Trait and Sickle Cell Anemia. Hematol. Am. Soc. Hematol. Educ. Program.

[42] Melo H.N., Stoots S.J.-M., Pool M.A., Carvalho V.O., Aragão M.L.D.C., Gurgel R.Q., Agyemang C., Cipolotti R. (2018). Objectively Measured Physical Activity Levels and Sedentary Time in Children and Adolescents with Sickle Cell Anemia. PLoS ONE.

[43] Marchese V., Rock K., Harpold A., Salazar A., Williams M., Shipper A.G. (2022). Physical Impairment and Function in Children and Adolescents with Sickle Cell Disease: A Systematic Review. Arch. Phys. Med. Rehabil..

[44] Liem R.I., Reddy M., Pelligra S.A., Savant A.P., Fernhall B., Rodeghier M., Thompson A.A. (2015). Reduced Fitness and Abnormal Cardiopulmonary Responses to Maximal Exercise Testing in Children and Young Adults with Sickle Cell Anemia. Physiol. Rep..

[45] Miller D.M., Winslow R.M., Klein H.G., Wilson K.C., Brown F.L., Statham N.J. (1980). Improved Exercise Performance After Exchange Transfusion in Subjects with Sickle Cell Anemia. Blood.

[46] Waltz X., Romana M., Lalanne-Mistrih M.-L., Machado R.F., Lamarre Y., Tarer V., Hardy-Dessources M.-D., Tressières B., Divialle-Doumdo L., Petras M. (2013). Hematologic and Hemorheological Determinants of Resting and Exercise-Induced Hemoglobin Oxygen Desaturation in Children with Sickle Cell Disease. Haematologica.

[47] Lester L.A., Sodt P.C., Hutcheon N., Arcilla R.A. (1990). Cardiac Abnormalities in Children with Sickle Cell Anemia. Chest.

[48] Hankins J.S., McCarville M.B., Hillenbrand C.M., Loeffler R.B., Ware R.E., Song R., Smeltzer M.P., Joshi V. (2010). Ventricular Diastolic Dysfunction in Sickle Cell Anemia Is Common but Not Associated with Myocardial Iron Deposition. Pediatr. Blood Cancer.

[49] Johnson M.C., Kirkham F.J., Redline S., Rosen C.L., Yan Y., Roberts I., Gruenwald J., Marek J., DeBaun M.R. (2010). Left Ventricular Hypertrophy and Diastolic Dysfunction in Children with Sickle Cell Disease Are Related to Asleep and Waking Oxygen Desaturation. Blood.

[50] Hammoudi N., Ceccaldi A., Haymann J.-P., Guedeney P., Nicolas-Jilwan F., Zeitouni M., Montalescot G., Lionnet F., Isnard R., Hatem S.N. (2022). Altered Cardiac Reserve Is a Determinant of Exercise Intolerance in Sickle Cell Anaemia Patients. Eur. J. Clin. Investig..

[51] Alsaied T., Niss O., Powell A.W., Fleck R.J., Cnota J.F., Chin C., Malik P., Quinn C.T., Taylor M.D. (2018). Diastolic Dysfunction Is Associated with Exercise Impairment in Patients with Sickle Cell Anemia. Pediatr. Blood Cancer.

[52] d’Humières T., Bouvarel A., Boyer L., Savale L., Guillet H., Alassaad L., de Luna G., Berti E., Iles S., Pham Hung d’Alexandry d’Orengiani A.L. (2024). Cardiac Diastolic Maladaptation Is Associated with the Severity of Exercise Intolerance in Sickle Cell Anemia Patients. Sci. Rep..

[53] MacLean J.E., Atenafu E., Kirby-Allen M., MacLusky I.B., Stephens D., Grasemann H., Subbarao P. (2008). Longitudinal Decline in Lung Volume in a Population of Children with Sickle Cell Disease. Am. J. Respir. Crit. Care Med..

[54] Lunt A., McGhee E., Sylvester K., Rafferty G., Dick M., Rees D., Height S., Thein S.L., Greenough A. (2016). Longitudinal Assessment of Lung Function in Children with Sickle Cell Disease. Pediatr. Pulmonol..

[55] Arigliani M., Gupta A. (2020). Management of Chronic Respiratory Complications in Children and Adolescents with Sickle Cell Disease. Eur. Respir. Rev..

[56] Miller A.C., Gladwin M.T. (2012). Pulmonary Complications of Sickle Cell Disease. Am. J. Respir. Crit. Care Med..

[57] Liem R.I., Nevin M.A., Prestridge A., Young L.T., Thompson A.A. (2009). Functional Capacity in Children and Young Adults with Sickle Cell Disease Undergoing Evaluation for Cardiopulmonary Disease. Am. J. Hematol..

[58] van Beers E.J., van der Plas M.N., Nur E., Bogaard H.-J., van Steenwijk R.P., Biemond B.J., Bresser P. (2014). Exercise Tolerance, Lung Function Abnormalities, Anemia, and Cardiothoracic Ratio in Sickle Cell Patients. Am. J. Hematol..

[59] Callahan L.A., Woods K.F., Mensah G.A., Ramsey L.T., Barbeau P., Gutin B. (2002). Cardiopulmonary Responses to Exercise in Women with Sickle Cell Anemia. Am. J. Respir. Crit. Care Med..

[60] Charlot K., Waltz X., Hedreville M., Sinnapah S., Lemonne N., Etienne-Julan M., Soter V., Hue O., Hardy-Dessources M.-D., Connes P. (2015). Impaired Oxygen Uptake Efficiency Slope and Off-Transient Kinetics of Pulmonary Oxygen Uptake in Sickle Cell Anemia Are Associated with Hemorheological Abnormalities. Clin. Hemorheol. Microcirc..

[61] Ogunsile F.J., Stewart K.J., Kanter J., Lanzkron S.M. (2022). An Evaluation of Cardiopulmonary Endurance and Muscular Strength in Adults Living with Sickle Cell Disease. Br. J. Haematol..

[62] Gouraud E., Connes P., Gauthier-Vasserot A., Faes C., Merazga S., Poutrel S., Renoux C., Boisson C., Joly P., Bertrand Y. (2021). Is Skeletal Muscle Dysfunction a Limiting Factor of Exercise Functional Capacity in Patients with Sickle Cell Disease?. J. Clin. Med..

[63] Merlet A.N., Féasson L., Bartolucci P., Hourdé C., Schwalm C., Gellen B., Galactéros F., Deldicque L., Francaux M., Messonnier L.A. (2020). Muscle Structural, Energetic and Functional Benefits of Endurance Exercise Training in Sickle Cell Disease. Am. J. Hematol..

[64] Ravelojaona M., Féasson L., Oyono-Enguéllé S., Vincent L., Djoubairou B., Essoue C.E., Messonnier L.A. (2015). Evidence for a Profound Remodeling of Skeletal Muscle and Its Microvasculature in Sickle Cell Anemia. Am. J. Pathol..

[65] Merlet A.N., Chatel B., Hourdé C., Ravelojaona M., Bendahan D., Féasson L., Messonnier L.A. (2019). How Sickle Cell Disease Impairs Skeletal Muscle Function: Implications in Daily Life. Med. Sci. Sports Exerc..

[66] Miller G.J., Serjeant G.R., Sivapragasam S., Petch M.C. (1973). Cardio-Pulmonary Responses and Gas Exchange during Exercise in Adults with Homozygous Sickle-Cell Disease (Sickle-Cell Anaemia). Clin. Sci..

[67] Mougin L., Riccetti M., Merlet A.N., Bartolucci P., Gellen B., Blervaque L., d’Humières T., Galactéros F., Emhoff C.-A.W., Féasson L. (2024). Endurance Training Improves Oxygen Uptake/Demand Mismatch, Metabolic Flexibility and Recovery in Patients with Sickle Cell Disease. Haematologica.

[68] Buchowski M.S., Townsend K.M., Williams R., Chen K.Y. (2002). Patterns and Energy Expenditure of Free-Living Physical Activity in Adolescents with Sickle Cell Anemia. J. Pediatr..

[69] Murray N., May A. (1988). Painful Crises in Sickle Cell Disease--Patients’ Perspectives. BMJ.

[70] Bartolucci P., Habibi A., Khellaf M., Roudot-Thoraval F., Melica G., Lascaux A.-S., Moutereau S., Loric S., Wagner-Ballon O., Berkenou J. (2016). Score Predicting Acute Chest Syndrome During Vaso-Occlusive Crises in Adult Sickle-Cell Disease Patients. eBioMedicine.

[71] Ploeger H.E., Takken T., de Greef M.H.G., Timmons B.W. (2009). The Effects of Acute and Chronic Exercise on Inflammatory Markers in Children and Adults with a Chronic Inflammatory Disease: A Systematic Review. Exerc. Immunol. Rev..

[72] Cerqueira É., Marinho D.A., Neiva H.P., Lourenço O. (2020). Inflammatory Effects of High and Moderate Intensity Exercise—A Systematic Review. Front. Physiol..

[73] Liem R.I., Onyejekwe K., Olszewski M., Nchekwube C., Zaldivar F.P., Radom-Aizik S., Rodeghier M.J., Thompson A.A. (2015). The Acute Phase Inflammatory Response to Maximal Exercise Testing in Children and Young Adults with Sickle Cell Anaemia. Br. J. Haematol..

[74] Sarray S., Saleh L.R., Lisa Saldanha F., Al-Habboubi H.H., Mahdi N., Almawi W.Y. (2015). Serum IL-6, IL-10, and TNFα Levels in Pediatric Sickle Cell Disease Patients during Vasoocclusive Crisis and Steady State Condition. Cytokine.

[75] Qari M.H., Dier U., Mousa S.A. (2012). Biomarkers of Inflammation, Growth Factor, and Coagulation Activation in Patients with Sickle Cell Disease. Clin. Appl. Thromb Hemost..

[76] Jilma B., Eichler H.G., Stohlawetz P., Dirnberger E., Kapiotis S., Wagner O.F., Schütz W., Krejcy K. (1997). Effects of Exercise on Circulating Vascular Adhesion Molecules in Healthy Men. Immunobiology.

[77] Nader E., Skinner S., Romana M., Fort R., Lemonne N., Guillot N., Gauthier A., Antoine-Jonville S., Renoux C., Hardy-Dessources M.-D. (2019). Blood Rheology: Key Parameters, Impact on Blood Flow, Role in Sickle Cell Disease and Effects of Exercise. Front. Physiol..

[78] Chatel B., Messonnier L.A., Bendahan D. (2018). Do We Have to Consider Acidosis Induced by Exercise as Deleterious in Sickle Cell Disease?. Exp. Physiol..

[79] Reinhart W.H., Gaudenz R., Walter R. (2002). Acidosis Induced by Lactate, Pyruvate, or HCl Increases Blood Viscosity. J. Crit. Care.

[80] Chatel B., Messonnier L.A., Hourdé C., Vilmen C., Bernard M., Bendahan D. (2017). Moderate and Intense Muscular Exercises Induce Marked Intramyocellular Metabolic Acidosis in Sickle Cell Disease Mice. J. Appl. Physiol..

[81] Hines P.C., Zen Q., Burney S.N., Shea D.A., Ataga K.I., Orringer E.P., Telen M.J., Parise L.V. (2003). Novel Epinephrine and Cyclic AMP-Mediated Activation of BCAM/Lu-Dependent Sickle (SS) RBC Adhesion. Blood.

[82] El Nemer W., Wautier M.-P., Rahuel C., Gane P., Hermand P., Galactéros F., Wautier J.-L., Cartron J.-P., Colin Y., Le Van Kim C. (2007). Endothelial Lu/BCAM Glycoproteins Are Novel Ligands for Red Blood Cell A4β1integrin: Role in Adhesion of Sickle Red Blood Cells to Endothelial Cells. Blood.

[83] Brousse V., Pondarre C., Arnaud C., Kamden A., de Montalembert M., Boutonnat-Faucher B., Bourdeau H., Charlot K., Grévent D., Verlhac S. (2020). One-Fifth of Children with Sickle Cell Anemia Show Exercise-Induced Hemoglobin Desaturation: Rate of Perceived Exertion and Role of Blood Rheology. J. Clin. Med..

[84] Campbell A., Minniti C.P., Nouraie M., Arteta M., Rana S., Onyekwere O., Sable C., Ensing G., Dham N., Luchtman-Jones L. (2009). Prospective Evaluation of Haemoglobin Oxygen Saturation at Rest and after Exercise in Paediatric Sickle Cell Disease Patients. Br. J. Haematol..

[85] Halphen I., Elie C., Brousse V., Bourgeois M.L., Allali S., Bonnet D., de Montalembert M. (2014). Severe Nocturnal and Postexercise Hypoxia in Children and Adolescents with Sickle Cell Disease. PLoS ONE.

[86] Sen C.K. (1995). Oxidants and Antioxidants in Exercise. J. Appl. Physiol..

[87] Lapoumeroulie C., Connes P., El Hoss S., Hierso R., Charlot K., Lemonne N., Elion J., Le Van Kim C., Romana M., Hardy-Dessources M.-D. (2019). New Insights into Red Cell Rheology and Adhesion in Patients with Sickle Cell Anaemia during Vaso-Occlusive Crises. Br. J. Haematol..

[88] Connes P., Simmonds M.J., Brun J.-F., Baskurt O.K. (2013). Exercise Hemorheology: Classical Data, Recent Findings and Unresolved Issues. Clin. Hemorheol. Microcirc..

[89] Charlot K., Romana M., Moeckesch B., Jumet S., Waltz X., Divialle-Doumdo L., Hardy-Dessources M.-D., Petras M., Tressières B., Tarer V. (2016). Which Side of the Balance Determines the Frequency of Vaso-Occlusive Crises in Children with Sickle Cell Anemia: Blood Viscosity or Microvascular Dysfunction?. Blood Cells. Mol. Dis..

[90] Waltz X., Hedreville M., Sinnapah S., Lamarre Y., Soter V., Lemonne N., Etienne-Julan M., Beltan E., Chalabi T., Chout R. (2012). Delayed Beneficial Effect of Acute Exercise on Red Blood Cell Aggregate Strength in Patients with Sickle Cell Anemia. Clin. Hemorheol. Microcirc..

[91] Balayssac-Siransy E., Connes P., Tuo N., Danho C., Diaw M., Sanogo I., Hardy-Dessources M.-D., Samb A., Ballas S.K., Bogui P. (2011). Mild Haemorheological Changes Induced by a Moderate Endurance Exercise in Patients with Sickle Cell Anaemia. Br. J. Haematol..

[92] Faes C., Balayssac-Siransy E., Connes P., Hivert L., Danho C., Bogui P., Martin C., Pialoux V. (2014). Moderate Endurance Exercise in Patients with Sickle Cell Anaemia: Effects on Oxidative Stress and Endothelial Activation. Br. J. Haematol..

[93] Grau M., Jerke M., Nader E., Schenk A., Renoux C., Collins B., Dietz T., Bizjak D.A., Joly P., Bloch W. (2019). Effect of Acute Exercise on RBC Deformability and RBC Nitric Oxide Synthase Signalling Pathway in Young Sickle Cell Anaemia Patients. Sci. Rep..

[94] Smith K.N., Baynard T., Fischbach P.S., Hankins J.S., Hsu L.L., Murphy P.M., Ness K.K., Radom-Aizik S., Tang A., Liem R.I. (2022). Safety of Maximal Cardiopulmonary Exercise Testing in Individuals with Sickle Cell Disease: A Systematic Review. Br. J. Sports Med..

[95] Radtke T., Crook S., Kaltsakas G., Louvaris Z., Berton D., Urquhart D.S., Kampouras A., Rabinovich R.A., Verges S., Kontopidis D. (2019). ERS Statement on Standardisation of Cardiopulmonary Exercise Testing in Chronic Lung Diseases. Eur. Respir. Rev..

[96] Charrin E., Aufradet E., Douillard A., Romdhani A., Souza G.D., Bessaad A., Faes C., Chirico E.N., Pialoux V., Martin C. (2015). Oxidative Stress Is Decreased in Physically Active Sickle Cell SAD Mice. Br. J. Haematol..

[97] Charrin E., Dubé J.J., Connes P., Pialoux V., Ghosh S., Faes C., Ofori-Acquah S.F., Martin C. (2018). Moderate Exercise Training Decreases Inflammation in Transgenic Sickle Cell Mice. Blood Cells Mol. Dis..

[98] Faes C., Charrin E., Connes P., Pialoux V., Martin C. (2015). Chronic Physical Activity Limits Blood Rheology Alterations in Transgenic SAD Mice. Am. J. Hematol..

[99] Gouraud E., Charrin E., Dubé J.J., Ofori-Acquah S.F., Martin C., Skinner S., Chatel B., Boreau A., Messonnier L.A., Connes P. (2019). Effects of Individualized Treadmill Endurance Training on Oxidative Stress in Skeletal Muscles of Transgenic Sickle Mice. Oxid. Med. Cell. Longev..

[100] Merlet A.N., Messonnier L.A., Coudy-Gandilhon C., Béchet D., Gellen B., Rupp T., Galactéros F., Bartolucci P., Féasson L. (2019). Beneficial Effects of Endurance Exercise Training on Skeletal Muscle Microvasculature in Sickle Cell Disease Patients. Blood.

[101] Junior J.A.d.A., Rossi D.A.A., Valadão T.F.C., Milan-Mattos J.C., Catai A.M., Sato T.d.O., Hueb J.C., Bazan S.G.Z., Hokama P.O.M., Hokama N.K. (2021). Cardiovascular Benefits of a Home-Based Exercise Program in Patients with Sickle Cell Disease. PLoS ONE.

[102] Liem R.I., Akinosun M., Muntz D.S., Thompson A.A. (2017). Feasibility and Safety of Home Exercise Training in Children with Sickle Cell Anemia. Pediatr. Blood Cancer.

[103] Gellen B., Messonnier L.A., Galactéros F., Audureau E., Merlet A.N., Rupp T., Peyrot S., Martin C., Féasson L., Bartolucci P. (2018). Moderate-Intensity Endurance-Exercise Training in Patients with Sickle-Cell Disease without Severe Chronic Complications (EXDRE): An Open-Label Randomised Controlled Trial. Lancet Haematol..

[104] Antonelli Rossi D.A., De Araujo Junior J.A., Luvizutto G.J., Bazan R., Salmazo P.S., Modolo G.P., Hueb J.C., Nunes H.R.d.C., Hokama N.K., Minicucci M.F. (2023). Effect of a Physical Exercise Program on the Inflammatory Response, Cardiac Functions, Functional Capacity, and Quality of Life in Patients with Sickle Cell Disease. J. Clin. Med..

[105] Almeida C.H.S.d., Reis L.F.d.F., Nascimento L.P.A.d.S., Soares A.R., Maioli M.C.P., Lopes A.J. (2021). Therapist-Oriented Home Rehabilitation for Adults with Sickle Cell Anemia: Effects on Muscle Strength, Functional Capacity, and Quality of Life. Hematology.

[106] Abd El-Kader S.M., Al-Shreef F.M. (2018). Impact of Aerobic Exercises on Selected Inflammatory Markers and Immune System Response among Patients with Sickle Cell Anemia in Asymptomatic Steady State. Afr. Health Sci..

[107] Pinto D.M.R., de Santana do Sacramento M., Santos P.H.S., Silva W.S., de Oliveira E.C., Gardenghi G., Ladeia A.M.T., Petto J. (2021). Physical Exercise in Sickle Cell Anemia: A Systematic Review. Hematol. Transfus. Cell Ther..

[108] Messonnier L.A., Riccetti M., Chatel B., Galactéros F., Gellen B., Rupp T., Féasson L., Bartolucci P. (2020). How to Implement Endurance Exercise Training in Sickle Cell Disease. Haematologica.

[109] Ojwaka M., González G.A., Sánchez S.S., Witmer L. (2025). Exercise and Sickle Cell Disease: A Systematic Review and Meta-Analysis. Ann. Fam. Med..

[110] De Lima F., Diaw M., Nader E., Carin R., Ducray M., Coly M.S., Charlot K., Marano M., Gallou-Guyot M., Diop S. (2026). Increasing Daily Step Counts Improves Physical Fitness, Reduces Pain and Arterial Stiffness in Sickle Cell Patients. Haematologica.

[111] Kasapis C., Thompson P.D. (2005). The Effects of Physical Activity on Serum C-Reactive Protein and Inflammatory Markers. JACC.

[112] Petersen A.M.W., Pedersen B.K. (2005). The Anti-Inflammatory Effect of Exercise. J. Appl. Physiol..

[113] Mattusch F., Dufaux B., Heine O., Mertens I., Rost R. (2000). Reduction of the Plasma Concentration of C-Reactive Protein Following Nine Months of Endurance Training. Int. J. Sports Med..

[114] Pedersen B.K., Hoffman-Goetz L. (2000). Exercise and the Immune System: Regulation, Integration, and Adaptation. Physiol. Rev..

[115] Pedersen B.K. (2017). Anti-Inflammatory Effects of Exercise: Role in Diabetes and Cardiovascular Disease. Eur. J. Clin. Investig..

[116] Gleeson M., Bishop N.C., Stensel D.J., Lindley M.R., Mastana S.S., Nimmo M.A. (2011). The Anti-Inflammatory Effects of Exercise: Mechanisms and Implications for the Prevention and Treatment of Disease. Nat. Rev. Immunol..

[117] Scheffer D.d.L., Latini A. (2020). Exercise-Induced Immune System Response: Anti-Inflammatory Status on Peripheral and Central Organs. Biochim. Biophys. Acta Mol. Basis Dis..

[118] Adamopoulos S., Parissis J., Kroupis C., Georgiadis M., Karatzas D., Karavolias G., Koniavitou K., Coats A.J., Kremastinos D.T. (2001). Physical Training Reduces Peripheral Markers of Inflammation in Patients with Chronic Heart Failure. Eur. Heart J..

[119] Benatti F.B., Pedersen B.K. (2015). Exercise as an Anti-Inflammatory Therapy for Rheumatic Diseases-Myokine Regulation. Nat. Rev. Rheumatol..

[120] Keikhaei B., Mohseni A.R., Norouzirad R., Alinejadi M., Ghanbari S., Shiravi F., Solgi G. (2013). Altered Levels of Pro-Inflammatory Cytokines in Sickle Cell Disease Patients during Vaso-Occlusive Crises and the Steady State Condition. Eur. Cytokine Netw..

[121] Kadoglou N.P.E., Iliadis F., Angelopoulou N., Perrea D., Ampatzidis G., Liapis C.D., Alevizos M. (2007). The Anti-Inflammatory Effects of Exercise Training in Patients with Type 2 Diabetes Mellitus. Eur. J. Cardiovasc. Prev. Rehabil..

[122] Ribeiro F., Alves A.J., Teixeira M., Miranda F., Azevedo C., Duarte J.A., Oliveira J. (2012). Exercise Training Increases Interleukin-10 after an Acute Myocardial Infarction: A Randomised Clinical Trial. Int. J. Sports Med..

[123] Collao N., Rada I., Francaux M., Deldicque L., Zbinden-Foncea H. (2020). Anti-Inflammatory Effect of Exercise Mediated by Toll-Like Receptor Regulation in Innate Immune Cells—A Review. Int. Rev. Immunol..

[124] Stewart L.K., Flynn M.G., Campbell W.W., Craig B.A., Robinson J.P., McFarlin B.K., Timmerman K.L., Coen P.M., Felker J., Talbert E. (2005). Influence of Exercise Training and Age on CD14+ Cell-Surface Expression of Toll-like Receptor 2 and 4. Brain. Behav. Immun..

[125] Wagener F.A., Feldman E., de Witte T., Abraham N.G. (1997). Heme Induces the Expression of Adhesion Molecules ICAM-1, VCAM-1, and E Selectin in Vascular Endothelial Cells. Proc. Soc. Exp. Biol. Med..

[126] Wagener F.A.D.T.G., Eggert A., Boerman O.C., Oyen W.J.G., Verhofstad A., Abraham N.G., Adema G., van Kooyk Y., de Witte T., Figdor C.G. (2001). Heme Is a Potent Inducer of Inflammation in Mice and Is Counteracted by Heme Oxygenase. Blood.

[127] Ghosh S., Adisa O.A., Chappa P., Tan F., Jackson K.A., Archer D.R., Ofori-Acquah S.F. (2013). Extracellular Hemin Crisis Triggers Acute Chest Syndrome in Sickle Mice. J. Clin. Investig..

[128] Demerath E., Towne B., Blangero J., Siervogel R.M. (2001). The Relationship of Soluble ICAM-1, VCAM-1, P-Selectin and E-Selectin to Cardiovascular Disease Risk Factors in Healthy Men and Women. Ann. Hum. Biol..

[129] Rohde L.E., Hennekens C.H., Ridker P.M. (1999). Cross-Sectional Study of Soluble Intercellular Adhesion Molecule-1 and Cardiovascular Risk Factors in Apparently Healthy Men. Arterioscler. Thromb. Vasc. Biol..

[130] Many G.M., Jenkins N.T., Witkowski S., Damsker J.M., Hagberg J. (2013). The Effects of Aerobic Training and Age on Plasma sICAM-1. Int. J. Sports Med..

[131] Zoppini G., Targher G., Zamboni C., Venturi C., Cacciatori V., Moghetti P., Muggeo M. (2006). Effects of Moderate-Intensity Exercise Training on Plasma Biomarkers of Inflammation and Endothelial Dysfunction in Older Patients with Type 2 Diabetes. Nutr. Metab. Cardiovasc. Dis. NMCD.

[132] Saetre T., Enoksen E., Lyberg T., Stranden E., Jørgensen J.J., Sundhagen J.O., Hisdal J. (2011). Supervised Exercise Training Reduces Plasma Levels of the Endothelial Inflammatory Markers E-Selectin and ICAM-I in Patients with Peripheral Arterial Disease. Angiology.

[133] Bjørnstad H.H., Bruvik J., Bjørnstad A.B., Hjellestad B.L., Damås J.K., Aukrust P. (2008). Exercise Training Decreases Plasma Levels of Soluble CD40 Ligand and P-Selectin in Patients with Chronic Heart Failure. Eur. J. Cardiovasc. Prev. Rehabil..

[134] Krakauer T. (1995). IL-10 Inhibits the Adhesion of Leukocytic Cells to IL-1-Activated Human Endothelial Cells. Immunol. Lett..

[135] Aufradet E., DeSouza G., Bourgeaux V., Bessaad A., Campion Y., Canet-Soulas E., Pialoux V., Chirico E.N., Chevrier A.-M., Godfrin Y. (2013). Hypoxia/Reoxygenation Stress Increases Markers of Vaso-Occlusive Crisis in Sickle SAD Mice. Clin. Hemorheol. Microcirc..

[136] Aufradet E., Douillard A., Charrin E., Romdhani A., De Souza G., Bessaad A., Faes C., Bourgeaux V., Chirico E.N., Canet-Soulas E. (2014). Physical Activity Limits Pulmonary Endothelial Activation in Sickle Cell SAD Mice. Blood.

[137] Diaw M., Connes P., Charlot K., Diop S., Gallou-Guyot M., Coly M.S., Carin R., Ducray M., Gadji M., Miyachi M. (2026). Relationship Between Daily Step Count, Biological Markers, and Clinical Outcomes in Patients with Sickle Cell Anemia: A Cross-Sectional Study. Eur. J. Haematol..

[138] Smith J.A., Martin D.T., Telford R.D., Ballas S.K. (1999). Greater Erythrocyte Deformability in World-Class Endurance Athletes. Am. J. Physiol..

[139] Suhr F., Brenig J., Müller R., Behrens H., Bloch W., Grau M. (2012). Moderate Exercise Promotes Human RBC-NOS Activity, NO Production and Deformability through Akt Kinase Pathway. PLoS ONE.

[140] Ernst E. (1987). Influence of Regular Physical Activity on Blood Rheology. Eur. Heart J..

[141] Brun J.F., Khaled S., Raynaud E., Bouix D., Micallef J.P., Orsetti A. (1998). The Triphasic Effects of Exercise on Blood Rheology: Which Relevance to Physiology and Pathophysiology?. Clin. Hemorheol. Microcirc..

[142] Romain A.-J., Brun J.-F., Varlet-Marie E., Raynaud de Mauverger E. (2011). Effects of Exercise Training on Blood Rheology: A Meta-Analysis. Clin. Hemorheol. Microcirc..

[143] Kilic-Toprak E., Ardic F., Erken G., Unver-Kocak F., Kucukatay V., Bor-Kucukatay M. (2012). Hemorheological Responses to Progressive Resistance Exercise Training in Healthy Young Males. Med. Sci. Monit. Int. Med. J. Exp. Clin. Res..

[144] Maiorana A., O’Driscoll G., Taylor R., Green D. (2003). Exercise and the Nitric Oxide Vasodilator System. Sports Med..

[145] Gliemann L., Nyberg M., Hellsten Y. (2014). Nitric Oxide and Reactive Oxygen Species in Limb Vascular Function: What Is the Effect of Physical Activity?. Free Radic. Res..

[146] Kleinbongard P., Schulz R., Rassaf T., Lauer T., Dejam A., Jax T., Kumara I., Gharini P., Kabanova S., Özüyaman B. (2006). Red Blood Cells Express a Functional Endothelial Nitric Oxide Synthase. Blood.

[147] Clarkson P.M., Thompson H.S. (2000). Antioxidants: What Role Do They Play in Physical Activity and Health?. Am. J. Clin. Nutr..

[148] Ohno H., Yahata T., Sato Y., Yamamura K., Taniguchi N. (1988). Physical Training and Fasting Erythrocyte Activities of Free Radical Scavenging Enzyme Systems in Sedentary Men. Eur. J. Appl. Physiol..

[149] Dokka S., Shi X., Leonard S., Wang L., Castranova V., Rojanasakul Y. (2001). Interleukin-10-Mediated Inhibition of Free Radical Generation in Macrophages. Am. J. Physiol. Lung Cell. Mol. Physiol..

[150] Gao Y., Tu D., Yang R., Chu C.-H., Hong J.-S., Gao H.-M. (2020). Through Reducing ROS Production, IL-10 Suppresses Caspase-1-Dependent IL-1β Maturation, Thereby Preventing Chronic Neuroinflammation and Neurodegeneration. Int. J. Mol. Sci..

[151] Barbeau P., Woods K.F., Ramsey L.T., Litaker M.S., Pollock D.M., Pollock J.S., Callahan L.A., Kutlar A., Mensah G.A., Gutin B. (2001). Exercise in Sickle Cell Anemia: Effect on Inflammatory and Vasoactive Mediators. Endothel. J. Endothel. Cell Res..

[152] Nystoriak M.A., Bhatnagar A. (2018). Cardiovascular Effects and Benefits of Exercise. Front. Cardiovasc. Med..

[153] Ferguson S., Gledhill N., Jamnik V.K., Wiebe C., Payne N. (2001). Cardiac Performance in Endurance-Trained and Moderately Active Young Women. Med. Sci. Sports Exerc..

[154] Tucker W.J., Beaudry R.I., Liang Y., Clark A.M., Tomczak C.R., Nelson M.D., Ellingsen O., Haykowsky M.J. (2019). Meta-Analysis of Exercise Training on Left Ventricular Ejection Fraction in Heart Failure with Reduced Ejection Fraction: A 10-Year Update. Prog. Cardiovasc. Dis..

[155] Linke A., Schoene N., Gielen S., Hofer J., Erbs S., Schuler G., Hambrecht R. (2001). Endothelial Dysfunction in Patients with Chronic Heart Failure: Systemic Effects of Lower-Limb Exercise Training. J. Am. Coll. Cardiol..

[156] Sachdev V., Machado R.F., Shizukuda Y., Rao Y.N., Sidenko S., Ernst I., St. Peter M., Coles W.A., Rosing D.R., Blackwelder W.C. (2007). Diastolic Dysfunction Is an Independent Risk Factor for Death in Patients with Sickle Cell Disease. J. Am. Coll. Cardiol..

[157] Xiong T., Bai X., Wei X., Wang L., Li F., Shi H., Shi Y. (2023). Exercise Rehabilitation and Chronic Respiratory Diseases: Effects, Mechanisms, and Therapeutic Benefits. Int. J. Chron. Obstruct. Pulmon. Dis..

[158] Armstrong M., Vogiatzis I. (2019). Personalized Exercise Training in Chronic Lung Diseases. Respirology.

[159] Li L.K., Cassim R., Perret J.L., Dharmage S.C., Lowe A.J., Lodge C.J., Russell M.A. (2023). The Longitudinal Association between Physical Activity, Strength and Fitness, and Lung Function: A UK Biobank Cohort Study. Respir. Med..

[160] Ohara D.G., Ruas G., Walsh I.A.P., Castro S.S., Jamami M. (2014). Lung Function and Six-Minute Walk Test Performance in Individuals with Sickle Cell Disease. Braz. J. Phys. Ther..

[161] Chatel B., Messonnier L.A., Barge Q., Vilmen C., Noirez P., Bernard M., Pialoux V., Bendahan D. (2018). Endurance Training Reduces Exercise-Induced Acidosis and Improves Muscle Function in a Mouse Model of Sickle Cell Disease. Mol. Genet. Metab..

[162] Diaw M., Coly M.S., Charlot K., Gallou-Guyot M., Miyachi M., Yoshida T., Gadji M., Diop S., Faye B.F., Mbengue A. (2025). Physical Activity, Vaso-Occlusive Crises and Pain in Patients with Sickle Cell Anaemia in Senegal. Br. J. Haematol..

[163] Telfer P., Coen P., Chakravorty S., Wilkey O., Evans J., Newell H., Smalling B., Amos R., Stephens A., Rogers D. (2007). Clinical Outcomes in Children with Sickle Cell Disease Living in England: A Neonatal Cohort in East London. Haematologica.

[164] Makani J., Cox S.E., Soka D., Komba A.N., Oruo J., Mwamtemi H., Magesa P., Rwezaula S., Meda E., Mgaya J. (2011). Mortality in Sickle Cell Anemia in Africa: A Prospective Cohort Study in Tanzania. PLoS ONE.

[165] Obeagu E.I., John A. (2025). Health Equity in Sickle Cell Disease: Overcoming Barriers to Care in Marginalized Communities. Ann. Med. Surg..

[166] De Bernardis S.C.Z., Olayinka O., Quraishi M.Z., Srivaths L.V., Brown D.L., Menon N.M., Stark J.M., Mosquera R.A., Patel A.P., Yadav A. (2026). Sickle Cell Disease Is an Inherent Risk for Asthma in a Sibling Comparison Study. Pediatr. Blood Cancer.

[167] Lettre G., Sankaran V.G., Bezerra M.A.C., Araújo A.S., Uda M., Sanna S., Cao A., Schlessinger D., Costa F.F., Hirschhorn J.N. (2008). DNA Polymorphisms at the BCL11A, HBS1L-MYB, and β-Globin Loci Associate with Fetal Hemoglobin Levels and Pain Crises in Sickle Cell Disease. Proc. Natl. Acad. Sci. USA.

[168] Waltz X., Romana M., Hardy-Dessources M.-D., Lamarre Y., Divialle-Doumdo L., Petras M., Tarer V., Hierso R., Baltyde K.-C., Tressières B. (2013). Hematological and Hemorheological Determinants of the Six-Minute Walk Test Performance in Children with Sickle Cell Anemia. PLoS ONE.

[169] Hsu L.L., Miller S.T., Wright E., Kutlar A., McKie V., Wang W., Pegelow C.H., Driscoll C., Hurlet A., Woods G. (2003). Alpha Thalassemia Is Associated with Decreased Risk of Abnormal Transcranial Doppler Ultrasonography in Children with Sickle Cell Anemia. J. Pediatr. Hematol. Oncol..

[170] Bernaudin F., Verlhac S., Chevret S., Torres M., Coic L., Arnaud C., Kamdem A., Hau I., Grazia Neonato M., Delacourt C. (2008). G6PD Deficiency, Absence of α-Thalassemia, and Hemolytic Rate at Baseline Are Significant Independent Risk Factors for Abnormally High Cerebral Velocities in Patients with Sickle Cell Anemia. Blood.

[171] Belisário A.R., Rodrigues C.V., Martins M.L., Silva C.M., Viana M.B. (2010). Coinheritance of α-Thalassemia Decreases the Risk of Cerebrovascular Disease in a Cohort of Children with Sickle Cell Anemia. Hemoglobin.

[172] Renoux C., Connes P., Nader E., Skinner S., Faes C., Petras M., Bertrand Y., Garnier N., Cuzzubbo D., Divialle-Doumdo L. (2017). Alpha-Thalassaemia Promotes Frequent Vaso-Occlusive Crises in Children with Sickle Cell Anaemia through Haemorheological Changes. Pediatr. Blood Cancer.

[173] Gardner K., Douiri A., Drasar E., Allman M., Mwirigi A., Awogbade M., Thein S.L. (2016). Survival in Adults with Sickle Cell Disease in a High-Income Setting. Blood.

[174] Guddal M.H., Stensland S.Ø., Småstuen M.C., Johnsen M.B., Zwart J.-A., Storheim K. (2019). Physical Activity and Sport Participation among Adolescents: Associations with Mental Health in Different Age Groups. Results from the Young-HUNT Study: A Cross-Sectional Survey. BMJ Open.

[175] Hoffmann M.D., Barnes J.D., Tremblay M.S., Guerrero M.D. (2022). Associations between Organized Sport Participation and Mental Health Difficulties: Data from over 11,000 US Children and Adolescents. PLoS ONE.

[176] Noordstar J.J., Hulzebos E.H.J., van der Ent C.K., Suijker M.H., Bartels M. (2023). Organized Sports Activities Are Safe for Children with Sickle Cell Disease: A Pilot Intervention Study. J. Pediatr. Hematol. Oncol..

[177] Sawka M.N., Montain S.J. (2000). Fluid and Electrolyte Supplementation for Exercise Heat Stress. Am. J. Clin. Nutr..

[178] Tewari S., Brousse V., Piel F.B., Menzel S., Rees D.C. (2015). Environmental Determinants of Severity in Sickle Cell Disease. Haematologica.

[179] Blumberg A.H., Ebelt S.T., Liang D., Morris C.R., Sarnat J.A. (2020). Ambient Air Pollution and Sickle Cell Disease-Related Emergency Department Visits in Atlanta, GA. Environ. Res..

[180] Wen T., Puett R.C., Liao D., Kanter J., Mittleman M.A., Lanzkron S.M., Yanosky J.D. (2024). Short-Term Air Pollution Levels and Sickle Cell Disease Hospital Encounters in South Carolina: A Case-Crossover Analysis. Environ. Res..

[181] Maclin K.M., Morgan C.J., Vilcassim R., Abadi A., Alishlash A., Pernell B.M. (2026). Pulmonary Function among Children and Young Adults with Sickle Cell Disease: The Potential Role of Air Pollution. J. Sick. Cell Dis..

[182] Kim S.R., Choi S., Keum N., Park S.M. (2020). Combined Effects of Physical Activity and Air Pollution on Cardiovascular Disease: A Population-Based Study. J. Am. Heart Assoc. Cardiovasc. Cerebrovasc. Dis..

[183] Raza W., Krachler B., Forsberg B., Sommar J.N. (2021). Air Pollution, Physical Activity and Ischaemic Heart Disease: A Prospective Cohort Study of Interaction Effects. BMJ Open.

[184] Hahad O., Kuntic M., Frenis K., Chowdhury S., Lelieveld J., Lieb K., Daiber A., Münzel T. (2021). Physical Activity in Polluted Air—Net Benefit or Harm to Cardiovascular Health? A Comprehensive Review. Antioxidants.

